# Research on Foot Contact State Detection Technology of Wheel-Legged Robot

**DOI:** 10.3390/s24123956

**Published:** 2024-06-18

**Authors:** Yaodong Wang, Meng Hong, Hui Chai, Yinglong Zhang, Guan Wang, Chaoqun Wu, Min Guo

**Affiliations:** 1 School of Mechanical and Electronic Engineering, Wuhan University of Technology, Wuhan 430070, China; yaodong@whut.edu.cn (Y.W.); hongmeng@whut.edu.cn (M.H.); zhangyinglong@whut.edu.cn (Y.Z.); 259607@whut.edu.cn (G.W.); chaoqunwu@whut.edu.cn (C.W.); 2 School of Control Science and Engineering, Shandong University, Jinan 250061, China; chaimax@sdu.edu.cn

**Keywords:** wheel-legged robot, foot-end contact, strain sensing, contact position, normal reaction force

## Abstract

The accurate perception of external environment information through the robot foot is crucial for the mobile robot to evaluate its ability to traverse terrain. Adequate foot-end contact signals can provide robust support for robot motion control and decision-making processes. The shape and uncertain rotation of the wheel-legged robot foot end represent a significant challenge to sensing the robot foot-end contact state, which current foot-end sensing schemes cannot solve. This paper presents a sensing method for the tire stress field of wheel-legged robots. A finite element analysis was conducted to study the deformation characteristics of the foot-end tire under force. Based on this analysis, a heuristic contact position estimator was designed that utilizes symmetrical deformation characteristics. Strain sensors, arranged in an array, extract the deformation information on the inner surface of the tire at a frequency of 200 Hz. The contact position estimator reduces the dimensionality of the data and fits the eigenvalues to the estimated contact position. Using support vector regression, the force estimator utilizes the estimated contact position and sensor signal to estimate the normal reaction force, designated as F_Z_. The sensing system is capable of detecting the contact position on the wheel circumference (with a root mean square error of 1.150°), as well as the normal force of 160 N on the Z axis (with a root mean square error of 6.04%). To validate the efficacy of the sensor detection method, a series of randomized and repeated experiments were conducted on a self-constructed test platform. This novel approach offers a promising avenue for perceiving contact states in wheel-legged robots.

## 1. Introduction

Obtaining an accurate and stable dynamic perception of the external environment is a crucial step in the future development of high-performance robots. Robots interact with their surroundings through various sensors, which facilitate stable and efficient operation. Currently, robots rely on non-contact sensors, such as cameras and LiDAR, to sense their environment. Combining the data obtained from these sensors allows the robot to acquire environmental information and make subsequent decisions. However, in instances of poor lighting or complex terrain, external sensors can be inaccurate or even fail. To ensure the robustness of robot operation, many researchers have begun to shift their focus to the field of robot contact sensing.

Legged robots interact with their surroundings through contact between the foot end and the ground. Contact sensing enables them to obtain haptic information from the external environment, including contact position and contact force magnitude, among other things [1]. This is achieved by using different foot-end contact sensors. Considering these sensors, Chuah et al. [2] developed a bimodal hemispherical footpad at MIT. This device employs a pressure sensor and a stress field approach to measure the contact position and normal and shear forces. Furthermore, the footpad system is robust upon impact, making it ideal for use in a range of applications. At the Max Planck Institute for Intelligent Systems, Felix et al. [3] developed a novel footTile sensor that determines reactive contact forces and centers of pressure between a robot and the ground in challenging terrains. Wilfred et al. [4] developed a haptic sensor for wheeled mobile robots by incorporating wheel-mounted acoustic waveguides throughout the circumference with the entire wheel week. This allows for the differentiation of diverse terrains, the identification and classification of various obstacles, and collision detection through contact localization. Wu et al. [5] developed a capacitive tactile sensor for a small hexapod robot with C-shaped rotating legs and integrated it into the robot’s feet. This allowed for the successful detection of the normal ground force while simultaneously enabling the construction of a terrain classifier and the implementation of terrain-based gait control. Research on haptic sensor technology has significant implications and potential for robotic applications. In legged robots, the shape of the foot end is flat, semi-circular, cylindrical, or bionic. However, wheel-legged robots have tire-shaped feet, and the wheels rotate continuously during wheeled motion. Therefore, haptic sensors cannot be effectively integrated into the foot end of a wheel-legged robot, thus posing a challenge for contact sensing.

The use of smart tires in vehicles can enhance their performance and increase drivers’ safety by providing reliable information about the tires’ operating conditions. One key challenge in the development of smart tires is embedding feasible and low-cost sensors to obtain data about the wheel–ground contact, such as vertical load, slip angle, etc. This challenge aligns with the direction of this study. To investigate the application of sensors for foot-end contact sensing in wheel-legged robots, this paper presents a detailed literature review on smart tires.

At present, tactile sensors for tires include built-in strain sensors [6,7], piezoresistive sensors [8,9,10], piezoelectric sensors [11], embedded accelerometers [12,13], rubber-based material substrates [14], fiber gratings [15], and embedded optical images [16]. Their main purpose is to determine the tire–ground contact relationship laterally by obtaining signals related to tire deformation. Previous research on smart tire sensors has shown that strain gauge sensors provide adequate contact sensing information through strain measurements. In addition, they are cost-efficient and reliable and have high accuracy for strain measurement under dynamic conditions.

The inability of wheel-legged robots to ignore the volume of foot-end tires precludes the existence of a directly usable sensing solution in the field of foot-end sensing for legged robots at this time. Conversely, in the field of smart tire sensing, the acquisition of contact signals in rolling modes, such as pendant force, tire pressure, and slippage force, facilitates the acquisition of contact information for wheel-legged robots. To address these challenges, this paper proposes a data fitting method that combines strain gauge sensor measurements, sensor signal feature extraction, and data fitting using a heuristic estimator and machine learning regression. In this method, strain gauge sensors are uniformly distributed on the inner surface of foot-end tires to obtain strain information. Polynomial regression and support vector regression are then applied to fit the sensor data and estimate the actual contact position and the magnitude of the vertical ground reaction force. The objective of this study is to determine the contact position and estimate the magnitude of the normal ground reaction force of a robot’s foot-end tire, as well as to investigate the potential of foot-end contact sensing in wheel-legged robots.

## 2. Materials and Methods

### 2.1. Sensor Design

**Figure 1:**
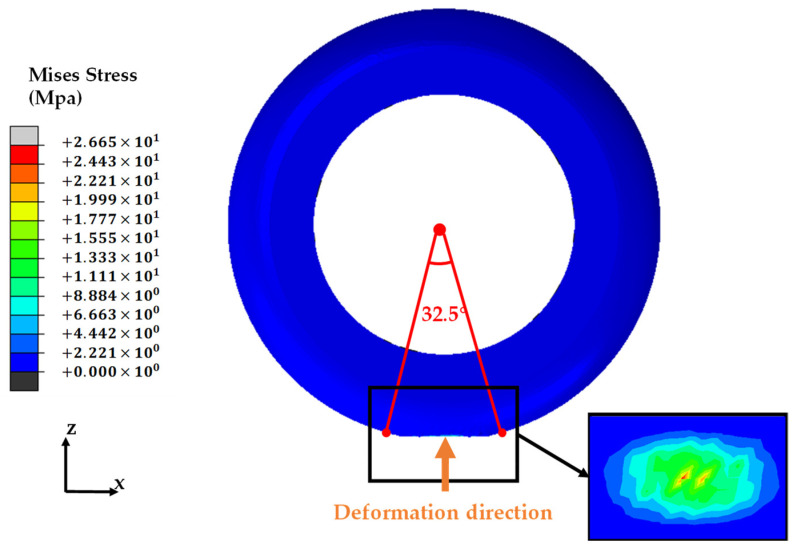
Visualization of stress distribution on the inner surface of foot-end tire.

For a preliminary exploration of force distribution on the inner surface of a robotic planter tire after deformation through contact with the ground, a finite element analysis of the tire model was carried out using ABAQUS 2020 simulation software. The model, as illustrated in Figure 1, comprises an analytic rigid body initially positioned at the base of the tire. This body was set to a constant velocity and employed to deform the mesh in compression by a prescribed amount. When the constraint in the Z-direction is released, a Z-directional load of 30 N is loaded. This is in the range of the contact force in the actual test. During the analysis process, the tire material was modeled as an isotropic hyperelastomeric material, as a linear elastic material is unable to accurately describe the material behavior, including the nonlinear elasticity of rubber. In this research, the Mooney–Rivlin material model was employed to elucidate the intrinsic structure of the rubber material, with the following parameters: C10 of 0.144 MPa; C01 of 0.036 MPa (C01 and C10 are the first and second parameters of the Mooney–Rivlin model); and bulk modulus D1 of 0, corresponding to the IRHD hardness value of 43 for the material properties. The figure depicts the results of the finite element analysis of the foot-end tire at an inflation pressure of 0.2 MPa. It is evident that when the tire’s outer side undergoes a certain deformation, the tire is subjected to the contact position of the inner surface. The Mises stress distribution is observed to exhibit a Gaussian distribution, with a uniform distribution in each sensing position (this study applied the typical area of 32.5° as an example). 

**Figure 2:**
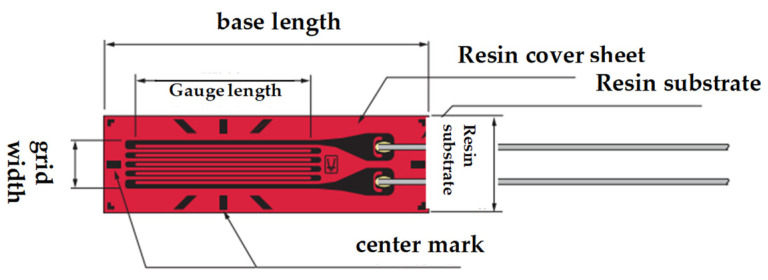
Strain gauge sensor.

Regarding sensor selection, piezoresistive, strain gauge, and fiber grating sensors are the most commonly utilized sensors for obtaining strain data. In this study, we considered the specific requirements of sensors for application in tires, including the need for high-quality sensors, ease of integration within the tire, and low complexity of signal acquisition systems. Thus, a uniaxial foil strain gauge sensor was used for strain detection, and its structure is depicted in Figure 2.

Furthermore, the characteristics of various strain gauge sensors were compared, and the relevant parameters are presented in Table 1. Among the different sensors, uniaxial and triaxial foil strain gauge sensors met our research needs. However, given the integration and feasibility challenges associated with the placement of multiple sensors in the tire, the uniaxial general-purpose foil strain gauge was the most suitable option.

We followed the methodology in previous research. In this study, strain gauge sensors were placed in the inner liner tread of the tire. With the application of external pressure, the internal resistance of the sensors increased or decreased linearly and proportionally. The following linear relationship was established:

(1) $$\frac{∆R}{R}=K_{S}\varepsilon$$

**Figure 3:**
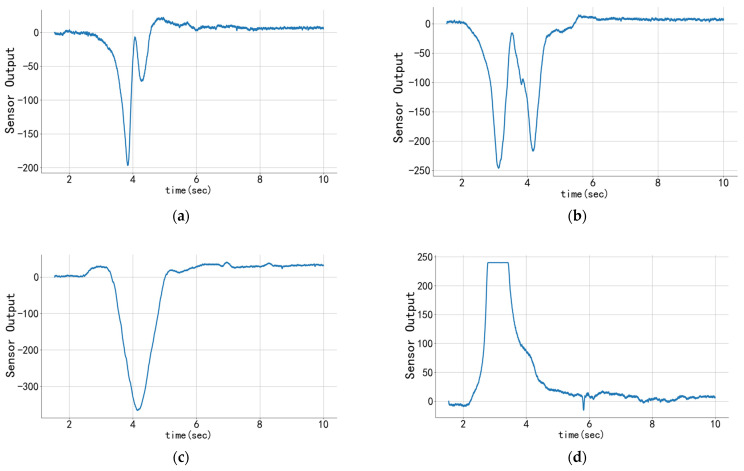
**The output of raw readings from sensors placed in different positions.** (**a**) Signal change in the tangential eccentric position; (**b**) signal change in the tangential central position; (**c**) signal change in the shear eccentric position; (**d**) signal change in the shear central position.

Tire strain measurements using uniaxial foil strain gauges are classified into tangential and shear measurements. To investigate the effect of different methods of attaching sensors to the inner surface of the tire on the extraction of contact data, we compared the measurement schemes for the centroid and eccentric positions in the tangential and shear directions, respectively, for individual measurements and gradually applied pressure to alter the contact position. The results are presented in Figure 3, where the sensor readings on the vertical coordinate are the 10-bit analog values obtained through the Arduino Due analog input interface, representing the raw output of the sensing system. Initialization zeroing was performed to ensure a comprehensive representation of the data, which is presented within the range of −512 to 512. Figure 3a,b illustrate the changes in sensor output signals placed at the center/eccentric position in the shear direction, while Figure 3c,d demonstrate the fluctuations in sensor output signals arranged in the center/eccentric position in the tangential direction. As shown in the figures, except for the Gaussian distribution at the shear-to-center position, the results of other detection positions indicate the presence of mutations around the contact position. This may affect the extraction of contact position information. Concurrently, the mutation is more pronounced in the tangential eccentric position, where more anomaly data can be extracted. 

**Figure 4:**
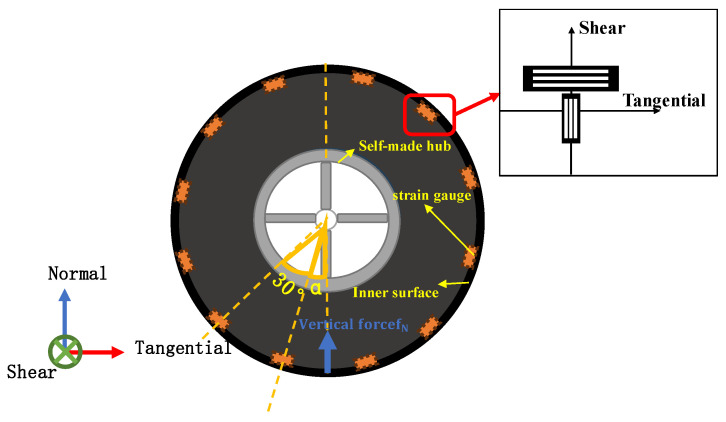
Schematic of sensor arrangement method.

Based on the aforementioned properties, the sensors were arranged in shear center and tangential eccentric positions. In the application of strain gauge sensors for smart tires, the sensors can typically be arranged in a single set to obtain adequate data with the rotation of the tire. However, in practice, since the robot will switch the wheel-leg motion mode according to changes in terrain, there are situations where rotation is limited or cannot occur. To obtain comprehensive data regarding the contact state, it is necessary to arrange enough sensors around the entire circumference of the tire. For measuring strain changes in the tire in the circumferential direction and ensuring the ability to detect the contact condition of the tire at any position in the tangential direction, it was initially determined that a combination of strain gauge sensors should be uniformly arranged in 12 groups along the tangential direction of the inner surface of the tire. The specific pasting arrangement method is illustrated in Figure 4. The contact position is defined by the angle α between the point of contact in the circumferential direction and the sensor.

### 2.2. Data Acquisition Module

**Figure 5:**
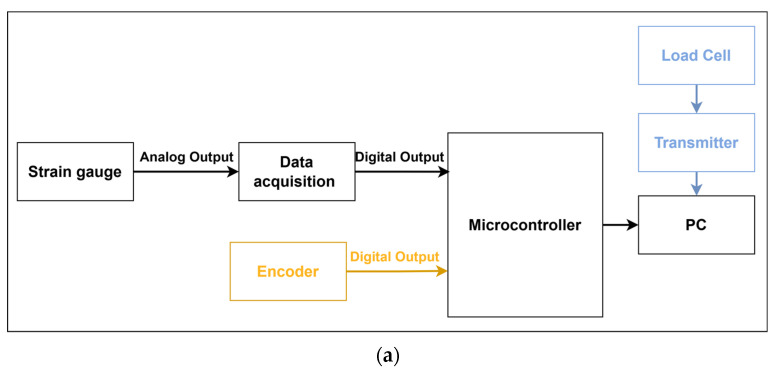

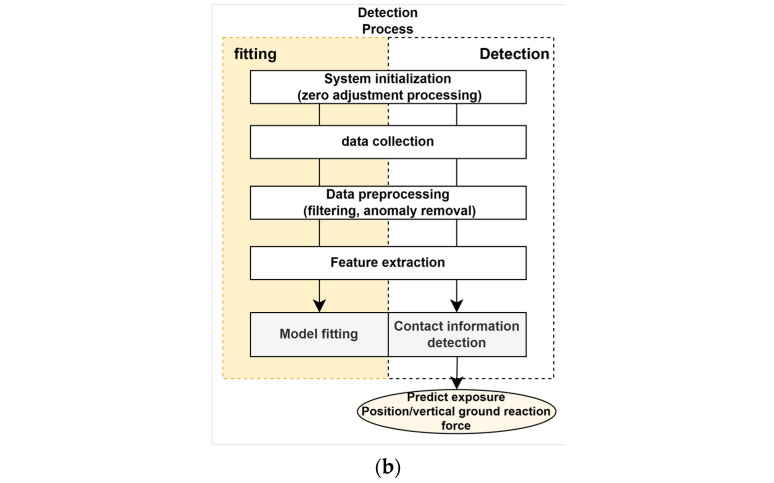
(**a**) The direction of the signal data in the hardware system is represented by the voltage variations from the strain gauge sensors, which are captured by the data acquisition module. These voltage variations are converted to analog data that can be used for model fitting and training by the analog-to-digital conversion module. The data are then sent to the PC for further data processing. (**b**) Sensor signal data acquisition and processing flow.

To acquire tire sensor data and subsequently transmit them to the signal processing system, a data acquisition circuit was used, comprising three modules: a control module, a communication module, and a data acquisition and conversion module. The controller was an Arduino Due microcontroller board with a 32-bit ARM architecture of the Atmel SAM3X8E CPU master chip, a clock frequency of up to 84 MHz, 54 digital input/output (IO) ports, and 12 analog input ports. A Bluetooth module was employed to transmit Bluetooth signals remotely to the robot’s upper computer by connecting the acquired signals to the Arduino Due board (the CPU was R7-5800H). The data acquisition and conversion module acquired strain signals and then converted small voltage signals to a range that could be received by the controller through two series-connected arithmetic circuits, finally converting them to analog signals for output through the ADC. During data acquisition, the actual contact position and the magnitude of the normal reaction force were collected by placing rotary encoders and load cells, respectively. The framework of the acquisition system is depicted in Figure 5a.

To minimize the impact of external interference on the acquired signals during sensor calibration, an artificial threshold was set to reduce the output of invalid data. As illustrated in Figure 5b, when the analog signal received by the host computer was below 5, the acquired data were designated as invalid data and set to zero. Additionally, in instances in which the sensor was not continuously utilized, its data were reset to zero by subtracting the initial reading at each initialization.

## 3. Experiments and Results

### 3.1. Contact Feature Estimation

**Figure 6:**
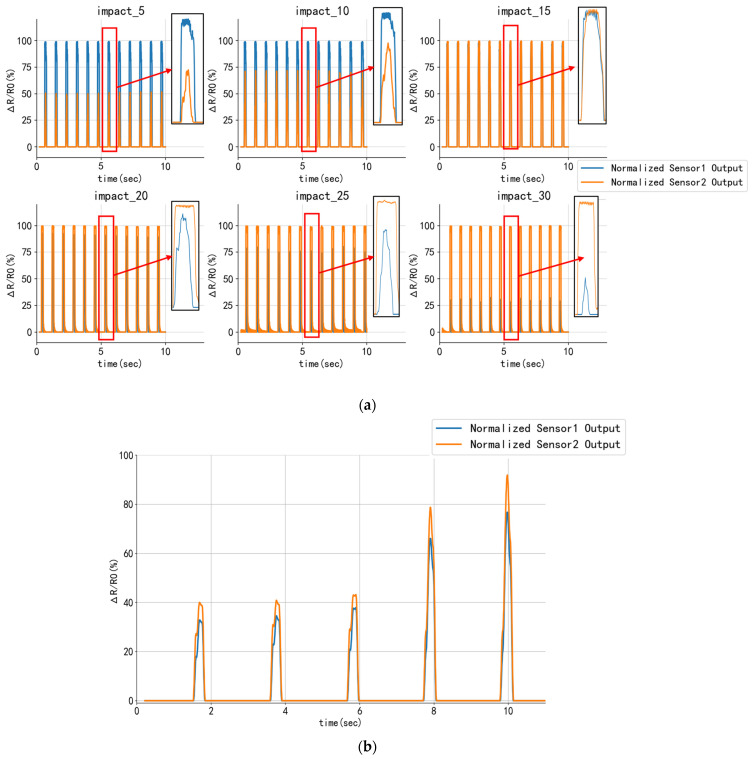
(**a**) Changes of sensor signals at different positions. As the contact position advances toward the two ends, the sensor signal difference will gradually increase, and an obvious symmetrical distribution characteristic appears. (**b**) The sensor signal reading changes under different contact force sizes are plotted. There is a positive correlation between the sensor signal readings when the given contact force is gradually increased.

To study sensor variations and their characteristics, different contact positions were arbitrarily chosen in the two sensor intervals under a specific normal load. Figure 6a depicts the variations in sensor signal intensity at the different positions, indicating regular fluctuations in the signals. For instance, when the contact position is at the positive center of the two sensors (at the 15° angle), the changing trend of the sensor signals is nearly identical; conversely, as the contact position gradually approaches the endpoints of the two sensors, the discrepancy between the two sensors increases.

To further elaborate this feature, the ratio *λ* of the difference in adjacent bimodal peaks and the larger of the two peaks is used as input, while the contact position, i.e., the angle of pinch *α*, is used as output for further data fitting. The ratio *λ* is denoted as follows:

(2) $$\lambda =\frac{S1-S2}{max(S1,S2)}$$

**Figure 7:**
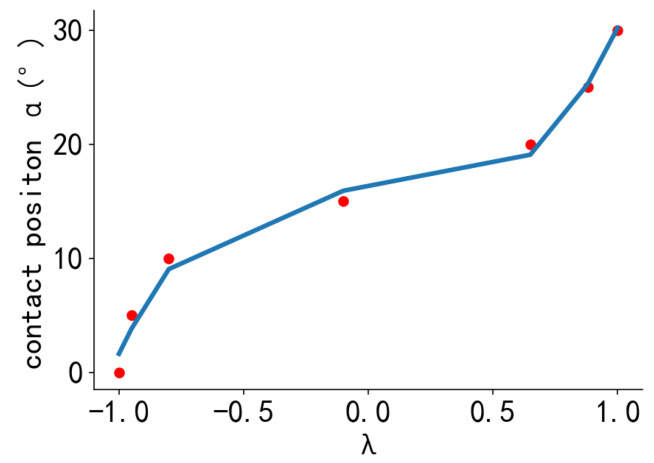
Relationship between contact position α and feature λ. The red dots represent the fitted source data and the blue lines represent the fitted results.

The λ values relative to *α*, obtained by calculating the sensor signals at different positions, are shown in Figure 7 (red dots). It can be observed that there is a functional relationship between the two variables, although it is not purely linear. Thus, choosing linear regression would result in underfitting. Among the different regression algorithms, polynomial regression is particularly well suited for modeling nonlinear relationships between input and output variables, as complex relationships that are difficult to model with linear regression can be delineated. Additionally, it has a more straightforward modeling process and does not require significant computational resources, rendering it a more suitable alternative for robots that require rapid real-time computation.

Polynomial regression can be considered an extension of linear regression that uses polynomial regression curves to fit nonlinear data using the following equation:

(3) $$f(x)=a_{n}x^{n}+a_{n-1}x^{n-1}+\ldots +a_{2}x^{2}+a_{1}x+a_{0}$$

where n denotes the order of the polynomial regression model. As the n value increases, the regression fitting results tend to converge to the true fit. Nevertheless, in practice, a higher order reflects the mapping relationship between inputs and outputs more realistically but leads to a decrease in the predictive ability for the test dataset primarily due to the insufficient generalization ability of the model. Conversely, a too-low order fails to accurately fit the regular relationship between inputs and outputs. Consequently, order selection is crucial in polynomial regression.

In this study, the model fitting was compared to the training set, and performance prediction was carried out on the test set to evaluate the performance of different polynomial order models. Our results indicate that the third-order polynomial model exhibited superior fitting performance and the lowest prediction error while also demonstrating strong model generalization capabilities. Therefore, the third-order polynomial model was selected for the prediction of the contact position signal. The equation used to determine the selected sensor signal’s eigenvalue *λ* relative to the contact position angle *α* (blue line in Figure 7) can be expressed as follows:

(4) $$\alpha \left(\lambda \right)=-0.7508\lambda^{3}+1.84\lambda^{2}-14.62\lambda +14.93$$

where *α* is the deviation angle used to characterize the contact position, and *λ* is the eigenvalue containing the sensor information

In the proposed sensing system, the magnitude of the normal reaction force changes with variations in the contact position and the normal load amount, governed by a highly complex nonlinear relationship that defies direct analytical modeling. As illustrated in Figure 6b, when progressively larger forces are applied at the same contact position, the signal value of each sensor increases simultaneously and in proportion. Given the regression method, achieving a more accurate fit of this nonlinear relationship is crucial for precise contact force detection. Thus, this method must be well suited for high-dimensional input scenarios and demonstrate improved generalization ability.

In comparison to linear regression and polynomial regression, SVR (support vector regression) also constructs a prediction model by learning the mapping relationship between the input and output data, which is well suited for nonlinear problems.

The model’s prediction accuracy is enhanced by adjusting the corresponding parameters. The fundamental distinction between SVR and other regression techniques is in the modeling method. SVR employs support vectors and kernel functions to generate a hyperplane, which minimizes prediction error and complexity [17]. This is achieved through convex optimization, thus ensuring the determination of the global optimal solution. The optimization problem can be expressed as follows:

(5) $$\underset{w,b}{\min}\frac{1}{2}{\left\|w\right\|}_{2}^{2}\;s.t.\left|y_{i}-(w^{T}x_{i}+b)\right|\leq \varepsilon ,\;i=1,2,\cdots ,N$$

where the input vector *x* is a 2D vector comprising measurements from the strain transducer signals S1 and S2, as well as the estimated value. The output scalar *y* represents the required vertical force. The vector $y_{i}$ is a 1D vector containing measurements at each point in the training dataset of *N* points. The allowable error *ε* is determined when the absolute value of the difference between the objective function and the true measurement $y_{i}$ is less than *ε*. For the SVR model developed in this study, the input vector x included the strain transducer signals S1 and S2, as well as the estimated value. The output scalar *y* was the estimated vertical force. The SVR model was initially trained using training data, and then its performance was evaluated using validation data.

**Figure 8:**
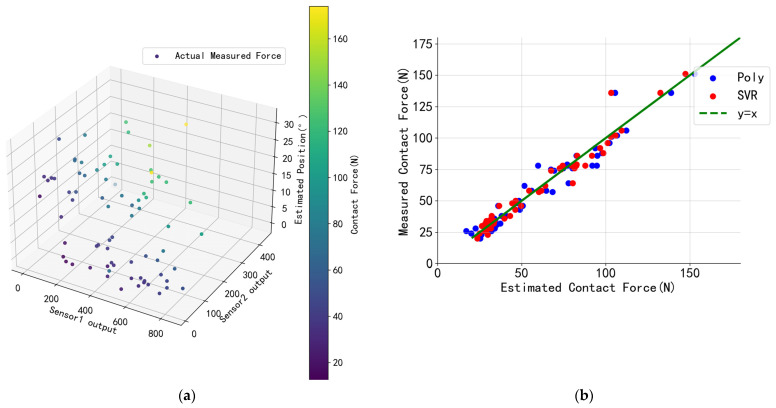
**Normal reaction force model predictions.** (**a**) Reflection of the data distribution used to train the SVR model. The input variable is the three features containing the raw output of sensors 1 and 2 and the estimated contact position, and the target variable is the magnitude of the normal contact force (indicated by the color bar). (**b**) Results of the comparison between the predicted and measured data. The closer the corresponding data points are to the y = x line, the closer the prediction is to the real value.

Contact positions were estimated using sensor signal data, and the estimated contact positions were then input into the SVR model as input values. The radial basis function (RBF) was selected as the kernel function. The regularization parameter C was adjusted to 6.5, and the tolerance ε was adjusted to 0.09, allowing for the prediction of the normal reaction force. Figure 8a illustrates the force distribution using the training and testing data, confirming the reliability of the training data interval and the consistency between the training and testing data. For the preliminary verification of the reliability of the selected model, a comparative analysis of the different fitted models was performed using the test set, and the results are presented in Figure 8b. The figure shows the results of the dataset using polynomial regression and support vector regression. The error analysis shows that polynomial regression yields an NRMSE of 3.43%, while the support vector regression yields an NRMSE of 2.91%. This suggests that the SVR model estimation is more accurate than the polynomial regression model.

### 3.2. Data Acquisition and Testing Equipment

Normalized experimental data necessitate the precise linear positioning of tires in both tangential and normal directions. The proposed sensing scheme involved a self-constructed test rig for acquiring and validating raw data. In the test platform, three-axis force transducers and absolute value encoders were employed to collect ground contact data in a real-world setting. A test stand was utilized to specify the force direction, enabling the tire motion and the adjustment of loading conditions along the set trajectory.

**Figure 9:**
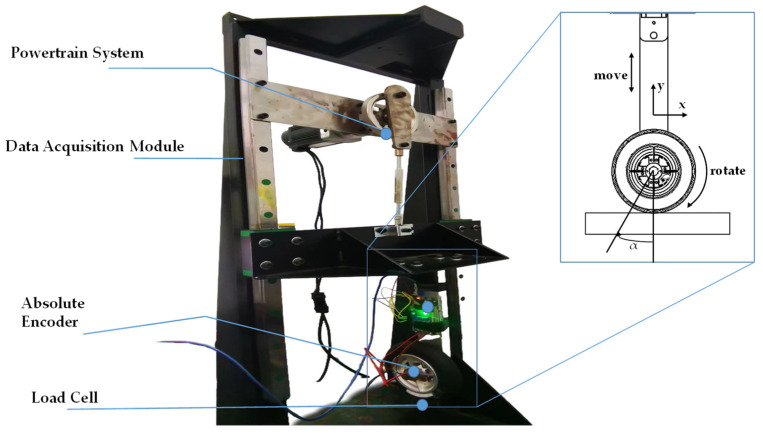
Sensing system testing setup.

In the experiment, the tire was rotated around the shear direction (X axis) from 0° to 30° in 5° increments to collect data from 1/12 of the contact area of the tire surface, as shown in Figure 9. At each contact location, sensor signals were acquired under the same load conditions using an orientation perpendicular to the load cell plane. As the tire sensor slowly moved through the prescribed trajectory, the Arduino Due lower unit read 10-bit analog values (0–1023) from the strain gauge sensor and the incremental encoder embedded in the inner surface of the tire through the interface at a sampling frequency of 200 Hz. At the same time, data were collected from the load cell and the rotary encoder at the bottom using the same frequency of 200 Hz and the data acquisition system in Figure 5a.

The operating conditions used to test the contact position and normal contact force are shown in Table 2, which contains the contact position and normal contact force test ranges, repeated contact test frequency, and tire pressure. 

### 3.3. Testing Results

To test the sensing system’s reliability and robustness for the detection of contact position, two sets of experiments were carried out based on the abovementioned test platform.

**Figure 10:**
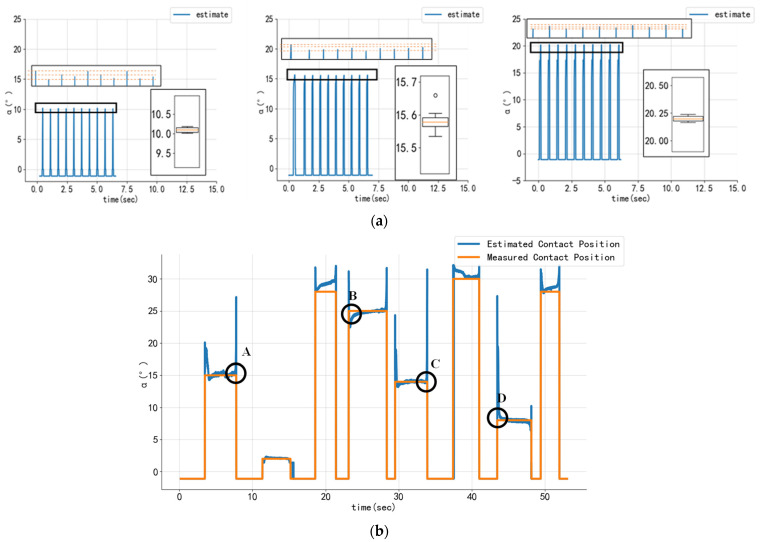
**Contact position detection results**. (**a**) The contact position detection results from the multiple impact test were plotted at 10°, 15°, and 20°, respectively. Each contact position result was plotted with a box plot to characterize the distribution of the estimated results. (**b**) Comparison of absolute encoder measurements from multiple static contact experiments with the estimated contact position (angle of divergence) of the array sensors. The estimated angle of contact position exhibits spikes since the sensors are no longer in contact with the ground, particularly at the point in time when contact is just being made (points A–D in the figure).

In the repeatability test, three contact positions (10°, 15°, and 20°) were selected, and 10 repeated compression experiments were performed at these positions. The error between the actual and the estimated positions was determined from the experimental results. The three test results are shown in Figure 10a. As can be seen, in the repetitive experiment, there is no large outlier in the contact position estimation, and the estimated root mean square error remains within 1°. This result is fully adequate for a wheel-legged robot requiring high-frequency touchdown and posture adjustment. Table 2 shows the root mean square error (RMS error) between the estimated and measured values of the validation dataset, which is used to determine the goodness of fit of the estimator.

The RMS error was used to assess the detection accuracy in both sets of experiments, and the results are shown in Table 3:

To test the robustness of the sensing system, we conducted a single set of static experiments at different random positions and compared the actual contact positions with the estimated positions. The test results are shown in Figure 10b. Larger spike bursts are observed in the estimated contact positions, corresponding to the times when the force on the foot end is very low or zero. This occurs when the sensor is barely or not at all in contact with the ground, and therefore the contact position cannot be estimated accurately. Although these spikes were included in all the RMS error calculations, they were only a small fraction of the data and therefore did not have a significant impact on performance. 

**Figure 11:**
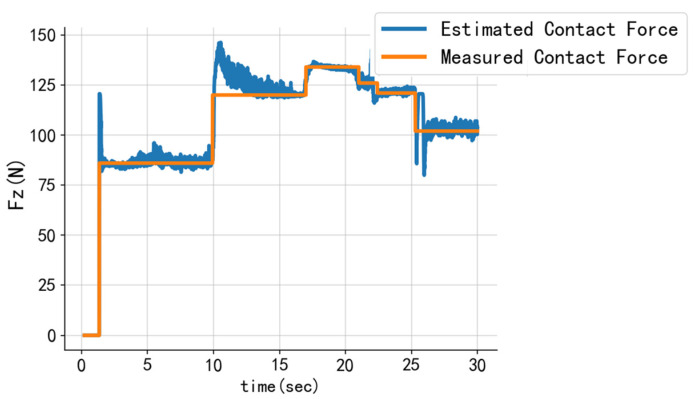
Comparing the measured results of the load cell with the estimated vertical force based on the sensors array, the normalized root mean squared error (NRMS) is 4.23%.

A randomized static test was considered the optimal method to evaluate the model’s performance. The results of the test are presented in Figure 11. The root mean square (RMS) error was 5.671 N, while the normalized RMS (NRMS) error was 4.23%. The results reveal that the observed fluctuations are not only a consequence of the relatively brief interval over which the magnitude of the contact force was altered but also due to the incorporation of the estimated contact position in the input data. In comparison to the estimation of contact position, the estimation results of the normal force exhibited greater noise. When the force was abruptly and rapidly altered, a significant offset was generated, and the system required a longer time to reach equilibrium. 

## 4. Conclusions and Discussion

In this study, we developed a contact sensing scheme for the foot-end tire of wheel-legged robots. The sensor is capable of detecting the stress distribution on the inner surface of the end-of-foot tire, as well as obtaining the contact position and normal reaction force in the foot end upon contact with the ground. This was achieved using heuristic-based polynomial fitting and support vector regression (SVR). To assess the efficacy of the proposed method for contact position estimation, repeated and random contact experiments were conducted. The results demonstrate that the root mean square (RMS) errors were 0.114°, 0.583°, and 0.203° at 10°, 15°, and 20° contact positions, respectively. Furthermore, the RMS error for the random position experiments was 1.150°, with a normalized root mean square (NRMS) error of 3.70%. A randomized force experiment was conducted to evaluate the model’s performance for contact force estimation, which yielded an RMS error of 5.671 N and an NRMS error of 4.23%.

The proposed sensing scheme offers several advantages. It is simple to install, lightweight, and exhibits low inertia. Additionally, its placement on the inner surface of the rubber tire eliminates the challenges commonly associated with short life and inertial noise sensitivity. Consequently, the system can rapidly detect contact positions and the normal reaction force. However, when the contact force was minimal, a significant discrepancy was observed. To address this problem, better machine learning methods can be employed for optimization. Additionally, robots frequently encounter diverse ground conditions, but the classification of multiple terrains was not addressed in this study. Future research will incorporate the detection of tangential upward stress changes to obtain tangential forces. Furthermore, the design of tire contact sensors and the acquisition of additional information from sensor signals, such as the distance traveled by the robot in rolling mode, will be investigated to provide more reliable information for slip detection and terrain classification.

## Figures and Tables

**Table 1:** Strain sensor performance parameters.

	BF350-3AA Single-Axis Foil Strain Gauge	BF350-3EB Full-Bridge Foil Strain Gauge	BF1K-3HA Half Bridge Biaxial Shear Strain Gauge	BF1K-3CA Tri-Axial Foil Strain Gauge
**Substrate size (length** × **width mm)**	6.8 × 4.2	10.5 × 8.5	9.7 × 6.7	10.2 × 10.2
**Dimensions of wire grid (length × width mm)**	3.0 × 3.0	3 × 3 (4 pieces)	2.8 × 5.5	3.0 × 2.1
**Maximum frequency (kHz)**	50	25	25	25
**Resistance (tolerance to nominal)**	350 ± 0.5 Ω	350 ± 0.5 Ω	1000 ± 3 Ω	1000 ± 3 Ω
**operating voltage**	5 V	5 V	5 V	5 V
**Sensitivity factor**	2.0 ± 1%	2.1 ± 1%	2.15 ± 1%	2.1 ± 1%
**Application areas**	Load sensors, pressure sensors, displacement sensors, acceleration sensors	Load sensors	Torque sensor	Load sensors, pressure sensors

**Table 2:** Test operation range.

Experimental Condition	Values
Contact position range	0–30°
Normal contact force range	0–160 N
Repeated contact frequency	1 Hz
Tire pressure	0.2 Mpa

**Table 3:** Contact position information detection results.

Contact Position (°)	Repetitive Experiment Group (°)	Random Experiment Group (°)
10°	0.114	1.150
15°	0.583	
20°	0.203	

## Data Availability

Data are contained within the article.
